# Development of a lateral flow recombinase polymerase amplification assay for rapid and visual detection of *Cryptococcus neoformans*/*C. gattii* in cerebral spinal fluid

**DOI:** 10.1186/s12879-019-3744-6

**Published:** 2019-02-04

**Authors:** Qinglin Ma, Jilong Yao, Shixin Yuan, Houming Liu, Ning Wei, Jianming Zhang, Wanshui Shan

**Affiliations:** 1 Institute of Maternity and Child Medical Research, Shenzhen Maternity and Child Healthcare Hospital, Southern Medical University, No.2004 Hongli Road, Shenzhen, 518028 Guangdong China; 2 Clinical Laboratory, Shenzhen Third People’s Hospital, No.29 Bulan Road, Shenzhen, 518112 Guangdong China; 3 Personnel Section, Shenzhen Maternity and Child Healthcare Hospital, Southern Medical University, No.2004 Hongli Road, Shenzhen, 518028 Guangdong China

**Keywords:** *Cryptococcus neoformans*, *Cryptococcus gattii*, Recombinase polymerase amplification, Lateral flow strips, Cerebral spinal fluid, Visual detection

## Abstract

**Background:**

For definitive diagnosis of cryptococcal meningitis, *Cryptococcus neoformans* and/or *C. gattii* must be identified within cerebral spinal fluid from the patients. The traditional methods for detecting *Cryptococcus* spp. such as India ink staining and culture are not ideal. Although sensitive and specific enough, detection of cryptococcal antigen polysaccharide has a high dose hook effect. Therefore, the aim of this study was to introduce a new rapid and simple detection method of *Cryptococcus neoformans* and *C. gattii* in cerebral spinal fluid.

**Methods:**

The lateral flow strips combined with recombinase polymerase amplification (LF-RPA) assay was constructed to detect the specific DNA sequences of *C. neoformans* and *C. gattii*. The detection limit was evaluated using serial dilutions of *C. neoformans* and *C. gattii* genomic DNA. The specificity was assessed by excessive amount of other pathogens genomic DNA. The optimal detection time and amplification temperature were also analyzed. The diagnostic parameters were first calculated using 114 clinical specimens and then compared with that of other diagnostic method. A brief analysis and comparison of different DNA extraction methods was discussed, too.

**Results:**

The LF-RPA assay could detect 0.64 pg of genomic DNA of *C. neoformans* per reaction within 10 min and was highly specific for *Cryptococcus* spp.. The system could work well at a wide range of temperature from 25 to 45 °C. The overall sensitivity and specificity were 95.2 and 95.8% respectively. As amplification template for LF-RPA assay, both cell lysates and genomic DNA produce similar experimental results.

**Conclusions:**

The LF-RPA system described here is shown to be a sensitive and specific method for the visible, rapid, and accurate detection of *Cryptococcus* spp. in cerebral spinal fluid and might be useful for clinical preliminary screening of cryptococcal meningitis.

## Background

The most common presentations of cryptococcosis are meningitis and meningoencephalitis [1]. Cryptococcal meningitis (CM) is a subacute meningoencephalitis and the most common cause of adult meningitis with very high mortality rates as well as vision and hearing loss in survivors [2–4]. The wide dispersal environmental fungi *Cryptococcus neoformans* and *C. gattii* are found particularly concentrated in soil and eucalyptus trees and responsible for the most cases of human cryptococcosis [3–5].

Early diagnosis and treatment of cryptococcosis reduces mortality. Lumbar puncture, also known as spinal tap, and cerebral spinal fluid (CSF) analysis should be performed in patients with suspected CM [3, 6]. For a definitive diagnosis of CM, *Cryptococcus* spp. must be identified within CSF from the patients [2, 3, 7]. India ink staining and culture are the traditional important methods for rapid detection of *Cryptococcus* spp. [6]. The sensitivity of India ink staining of the CSF is up to 70–90%, which tends to be lower in HIV-negative patients, but this value is dependent on both the fungal burden and operator [2–4, 8, 9]. The definitive diagnosis of CM relies on culture on standard Sabouraud dextrose agar (SDA) or using routine and automated culture systems inoculated with CSF incubated at 30 °C [4, 10]. However, culture may be negative if exposure to antifungal therapy or in non-HIV CM and might need longer incubation periods up to several weeks [8]. Serological diagnosis of CM, such as latex agglutination, enzyme-linked immunosorbent assays and lateral flow assay, relies usually on specific monoclonal antibodies to detect cryptococcal antigen polysaccharide (CrAg). Although detection of CrAg has demonstrated good sensitivity and specificity [11–15], extremely high concentrations of CrAg can yield negative test results in extreme instances, known as high dose hook effect.

Recently, recombinase polymerase amplification (RPA), an isothermal in vitro nucleic acid amplification technique, appeared as a novel molecular technology for simple, robust (less sensitive to inhibitors), rapid, reliable, and low-resource diagnostics [16–19]. At present, RPA combined with lateral flow strips (LF-RPA assay) has been successfully used for the rapid and visual detection of several pathogens including parasites, viruses, and bacteria [18–21]. In this study, we have assessed the performance of LF-RPA assay for detecting genomic DNA of *C. neoformans* and *C. gattii* in clinical CSF samples from patients.

## Methods

### Primer and probe design

To establish a nucleic acid-based detection method, the starting point is to identify the research target [22]. The internal transcribed spacer (ITS) sequences of ribosomal RNA gene are highly variable and useful for species differentiation [23–25]. A total of 139 available ITS sequences of *C. neoformans* or *C. gattii* were downloaded from the GenBank® database (https://www.ncbi.nlm.nih.gov/genbank/). DNAMAN software (Lynnon LLC., California, USA) was used to obtain the consensus sequence by multiple sequence alignment. Primer and probe for LF-RPA assay were designed based on the consensus sequence according to the guidelines of TwistAmp® DNA amplification kit (TwistDx Ltd., UK). The optimal primer-probe combination was obtained by screening via the basic local alignment search tool BLASTN (https://blast.ncbi.nlm.nih.gov/Blast.cgi) and actual testing. All oligonucleotides were synthesized by Beijing AuGCT DNA-SYN Biotechnology Co. Ltd. (Beijing, China) and shown in Table 1.

**Table 1:** Oligonucleotide primers and probe for LF-RPA assay

Name	Sequence (5’→3’)
CM ITS P4	FITC-TACACAAACTTCTAAATGTAATGAATGTAATC(H)TATTATAACA ATAATAAA-P
CM ITS F4	TGAACTGTTTATGTGCTTCGGCACGTTTTAC
CM ITS R4	Biotin-TCGATGTGGAAGCCAAGAGATCCGTTGTTG

CM: cryptococcal meningitis; ITS: internal transcribed spacer of ribosomal RNA gene; F: forward primer; R: reverse primer; P: probe; FITC: fluorescein isothiocyanate; H: tetrahydrofuran spacer; P: 3’ phosphate to block elongation.

### Clinical specimens and strains

To evaluate the diagnostic validity of our LF-RPA method, 114 CSF specimens (one sample from one patient with similar clinical symptoms to CM) were collected from Shenzhen Third People’s Hospital. At the time of the experiment, researchers did not know the pathogenic biological status of the above-mentioned samples. The results of LF-RPA assay and “CrAg Lateral Flow Assay” were compared to that of culture and/or India ink staining. Diagnostic parameters such as the overall specificity and sensitivity were evaluated using the free “diagnostic test evaluation calculator” (https://www.medcalc.org/calc/diagnostic_test.php) and expressed as percentages in Table 3.

India ink staining and “CrAg Lateral Flow Assay” were performed using the “*Cryptococcus neoformans* stain kit” (BA4042, Baso diagnostics, Inc. Zhuhai, China) and “Cryptococcal Antigen Lateral Flow Assay Kit” (CR2003, IMMY, Inc. Oklahoma, USA), respectively, according to the product instructions.

Culture was conducted at 25 °C and 37 °C for 2–4 weeks using the “BD BACTEC™ FX Blood Culture System” (BD Diagnostics, New Jersey, USA) and standard Sabouraud dextrose agar (Oxoid Limited, Hampshire, UK) [6, 10]. The positive cultures were screened for *Cryptococcus* spp. using the “MALDI Biotyper Systems” (Bruker Daltonik GmbH, Bremen, Germany).

In addition, clinical isolates were collected from Shenzhen Third People’s Hospital and the standard strains were bought from the American Type Culture Collection (ATCC, Manassas, Virginia, USA).

### DNA extraction

DNA extraction was conducted using glass beads (CapitalBio, Beijing, China) and boiling method as earlier reported with a little revision [26–28]. Briefly, a bit of sediments of CSF after centrifugation or fresh colonies were suspended in 50 μl of 1× TE buffer (10 mM Tris-HCl, 1 mM EDTA, pH 8.0) within an extraction tube and incubated at 95 °C in a boiling water-bath for 5 min. Then the total DNA was isolated by vortexing at maximum speed in an Extractor™ 36 (CapitalBio) for 5 min. After centrifuged at 10000 g for 5 min, the supernatant of the lysate containing gemonic DNA was separated for follow-up tests. This is “glass beads method” for sample cell lysate.

In order to meet the special needs of Fig. 4, we have further purified the supernatant of the lysate with “TIANamp Yeast DNA Kit” (TIANGEN Biotech, Beijing, China) starting from step 7 of the operating manual. This is “spin columns method” for DNA extraction.

DNA concentrations were accurately measured using the “Qubit™ 3 Fluorometer” (Q33216, Thermo Fisher Scientific, Wilmington, USA). All genomic DNA and sample lysate were kept in − 80 °C to preserve, and avoid repeated freeze-thaw cycles.

### RPA assay

RPA assay was carried out with 5 μl of template according to the operating manual of “TwistAmp® nfo kit” (TwistDx Ltd., UK) and the protocol previously described [20]. The “thermal cycler instrument” (HybriBio Ltd., Guangdong, China) was used to perform the reaction at 39 °C for half an hour. Heated lids should be switched off before start. After the first 4 min of incubation, the reaction tubes were vortexed and spun again to improve the amplification efficiency.

For visual analysis with Milenia® Genline Hybridetect-1 strips (Milenia Biotec GmbH, Germany), amplification product was diluted 1/10 with HybriDetect assay buffer inside a class II biosafety cabinet in product analysis room. Dipsticks were directly dipped into 50 μl of diluents at room temperature and the visual result should be observed within 5 min. If only the control band appears, it is considered to be negative result. If both the test and control bands display simultaneously, it is a positive result. If the control band is not visible after the incubation period, the result is invalid. The test must be repeated with a new dipstick. A piece of A4 paper was used to paste these dried dipsticks and then scanned by HP brother scanner (MFC-8535DN, Guangzhou, China).

### Assessment of LF-RPA performance

**Fig. 1:**
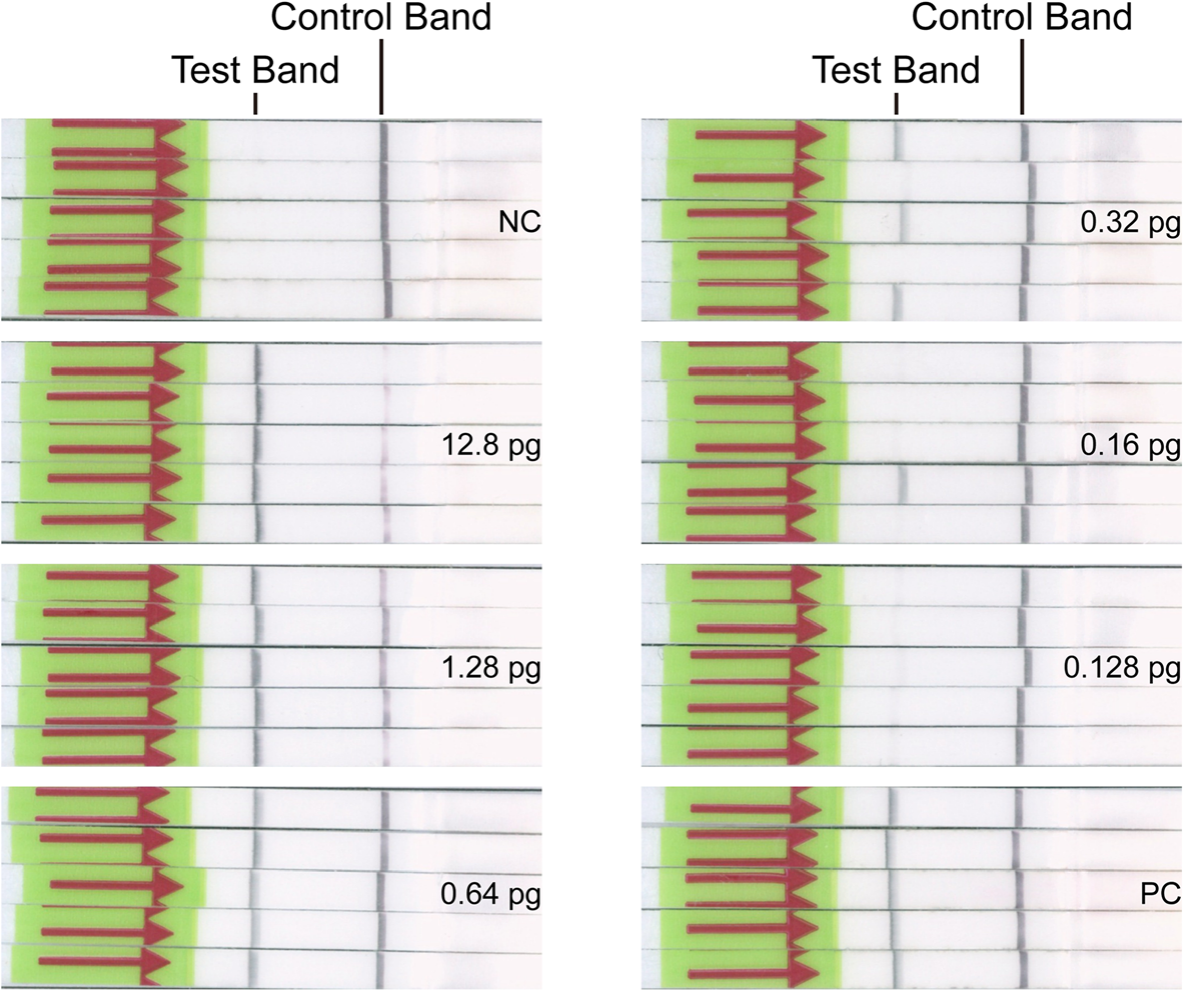
Evaluation of the detection threshold. Different quantity of genomic DNA of *C. neoformans var. grubii* H99 (ATCC 208821), shown on the right side, was used as templates. The LF-RPA assay could detect as low as 0.64 pg of genomic DNA of *C. neoformans* per test. pg: picogram; NC: negative control; PC: positive control

For evaluation of the detection limit (the lowest quantity of template for positive result), dilutions of genomic DNA of *C. neoformans var. grubii* H99 (ATCC 208821) to 12.8 pg, 1.28 pg, 0.64 pg, 0.32 pg, 0.16 pg and 0.128 pg per μl was prepared in nuclease-free water and 1 μl was used each reaction. LF-RPA assay was carried out in 5 replicates for each concentration gradient. The tests were repeated three times under the same conditions. One example of the results was presented in Fig. 1 and Fig. 4a.

**Fig. 2:**
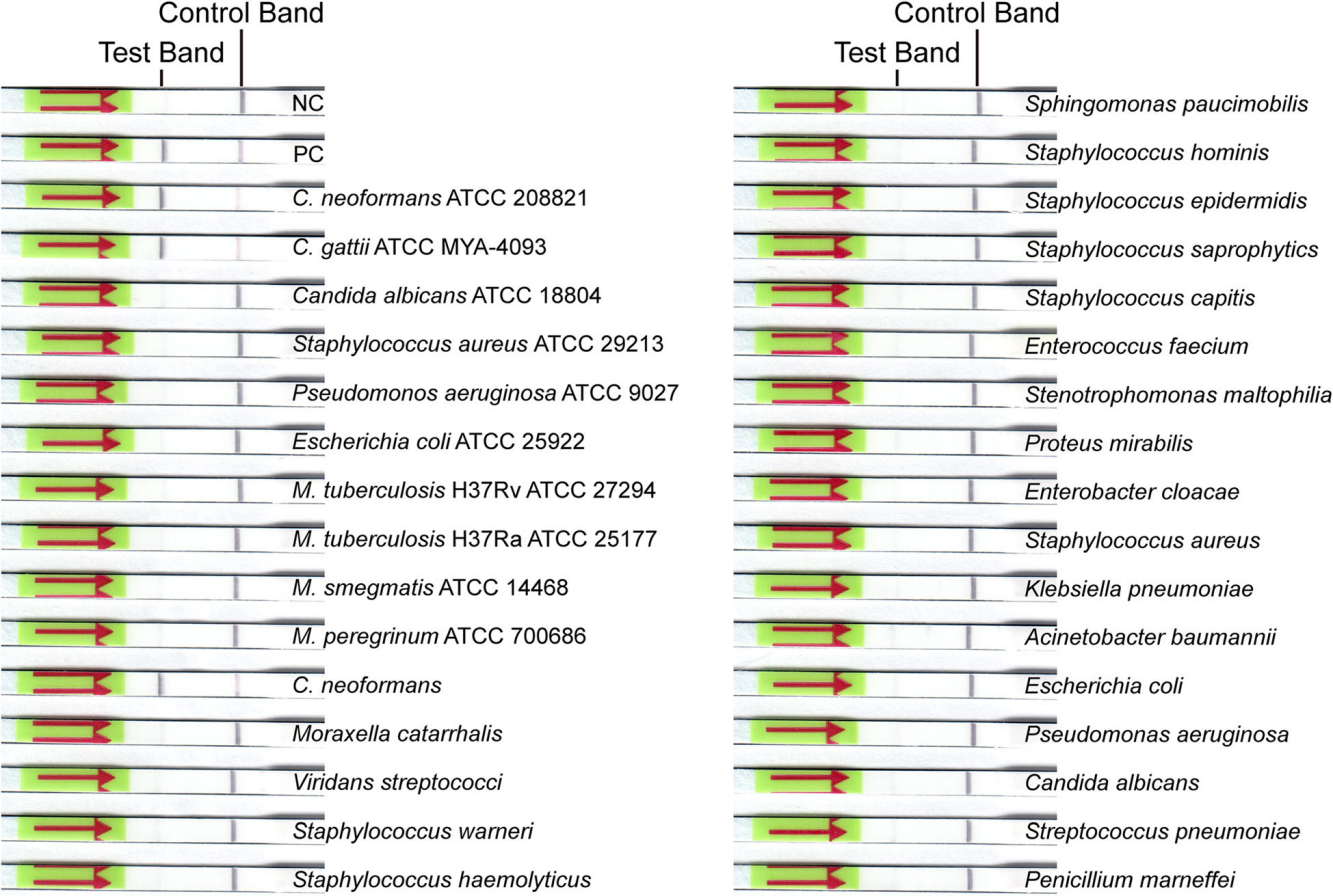
Cross-reactivity analysis. Excessive amount of genomic DNA from a variety of different pathogens including bacteria and fungi was used as templates. Cross-reaction with any other pathogens was not detected. NC: negative control; PC: positive control

To analyze the specificity of LF-RPA assay, at least 20 ng genomic DNA extracted from several pathogenic microorganisms which names were shown in Fig. 2 and Fig. 4b were used as templates each reaction.

The optimal amplification time was measured at 39 °C and terminated at different incubation time points ranging from 0 to 40 min by immediately placing on ice and diluting with HybriDetect assay buffer.

The optimal incubation temperature of RPA reaction was determined at 4, 10, 15, 20, 25, 30, 35, 40, 45 and 50 °C, respectively.

## Results

### Screening of optimal primer-probe combination

Four candidate primer-probe combinations were screened for the best performance under the same conditions. Primer-probe combination that produced clearly visible bands fastest and had no cross-reactions with other pathogens was chosen for subsequent evaluations. The final sequences of primers and probe are listed in Table 1.

### Detection limit

The detection threshold of LF-RPA assay was measured using serial dilutions of genomic DNA of *C. neoformans var. grubii* H99 (ATCC 208821) and *C. gattii* (ATCC MYA-4093). Five repetitive samples were analyzed for each concentration gradient (Table 2 & Fig. 1). One example of the results of three repeated experiments is shown in Fig. 1. Clear test and control bands appeared on each strip with greater than or equal to 0.64 pg of genomic DNA, while partial faint test bands or only control bands on other dipsticks (Fig. 1). Comprehensive five results per concentration gradient of three repeated experiments suggested that the LF-RPA assay could detect as low as 0.64 pg of genomic DNA of *C. neoformans* per reaction, and 1.016 pg of *C. gattii*.

**Table 2:** Calculated the detection limit of LF-RPA assay

DNA amount (pg/reaction)	NC	12.8	1.28	0.64	0.32	0.16	0.128	PC
*C*. *neoformans*	0/5^a^	5/5^a^	5/5^a^	5/5^a^	3/5^a^	1/5^a^	0/5^a^	5/5^a^
DNA amount (pg/reaction)	NC	20.32	2.032	1.016	0.508	0.254	0.2032	PC
*C*. *gattii*	0/5^a^	5/5^a^	5/5^a^	5/5^a^	4/5^a^	1/5^a^	0/5^a^	5/5^a^

^a^The number of positive samples/the total number of samples tested by LF-RPA assay. NC: negative control; PC: positive control.

### Cross-reactivity analysis

To evaluate the detection specificity of the established LF-RPA assay, cross-reactions were performed with excessive amount of genomic DNA from a variety of different pathogens as templates. As shown in Fig. 2, only the *Cryptococcus* spp. dipsticks including *C. neoformans* and *C. gattii* displayed a solid positive test band, while no signals were observed on the other strips even other fungi’s DNA as template. The results indicated that the primer-probe combination designed for the LF-RPA reactions was highly specific to its corresponding targets. It’s also suggested that the described LF-RPA assay was able to detect several different types of *Cryptococcus* strains, whether the clinical isolates or standard strains.

### Optimal amplification time and temperature

**Fig. 3:**
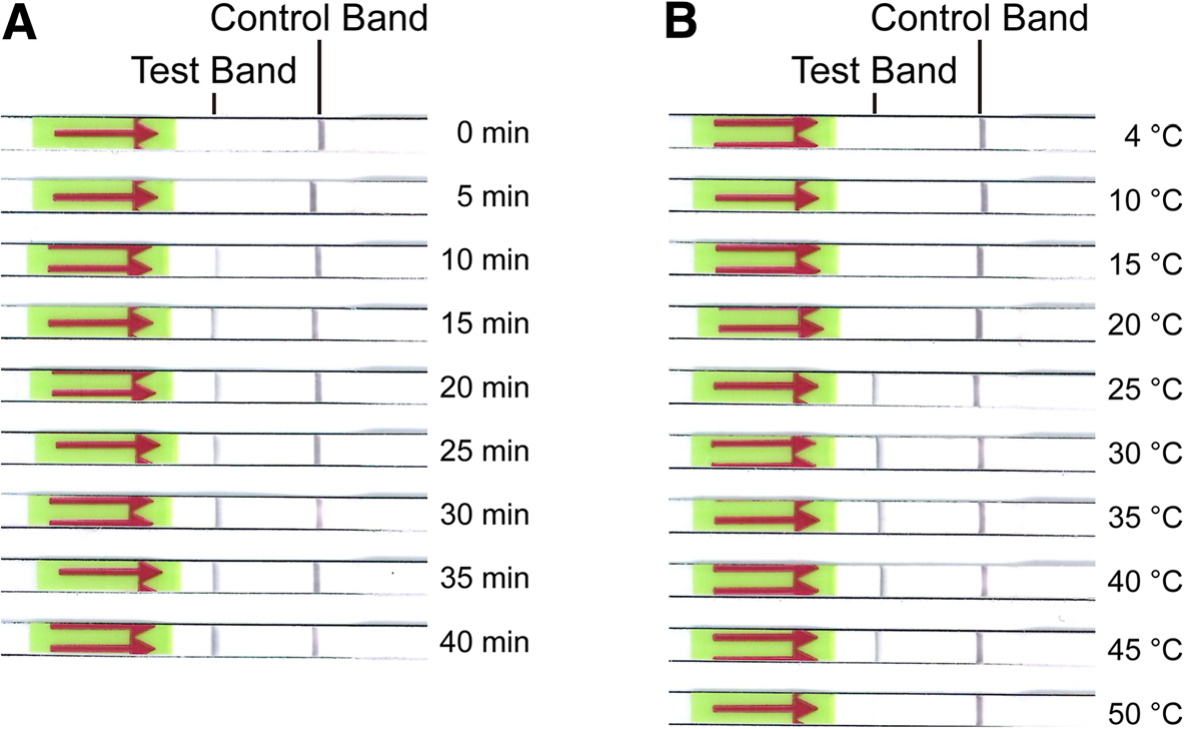
Analysis of optimal amplification time and temperature. The amplification time **(a)** and temperature **(b)** were shown on the right. 0.64 pg of genomic DNA of *C. neoformans var. grubii* H99 (ATCC 208821) was used as templates. **a** amplification time. A clear test band appeared and became more solid as time extended to 10 min or more. **b** amplification temperature. An obvious test band could be seen over a wide range of temperature from 25 to 45 °C.

To determine the optimal amplification time and temperature, the strips were incubated for 5 min at room temperature as earlier literatures reported [20, 29, 30] and 0.64 pg of genomic DNA of *C. neoformans var. grubii* H99 (ATCC 208821) was used as template. As the results shown in Fig. 3a, a clear test band could be observed and became more solid as time extended to 10 min or more. It means that the amplification time is at least 10 min for effective observation. The optimal amplification temperature for LF-RPA reactions was determined by using different temperature settings. As we can see in Fig. 3b, test bands clearly appeared on the strips over a wide range of temperature from 25 to 45 °C as previous study [20, 29], suggesting that the amplification reaction of LF-RPA assay is not sensitive to temperature gradients within a certain range.

### Analysis of clinical specimens

In order to determine the diagnostic applicability, the LF-RPA assay and “CrAg Lateral Flow Assay” were compared to the gold standard diagnoses of cryptococcal meningitis (culture and/or India ink staining). These studies contained a mix of both prospective and retrospective specimens. A summary of the data collected was included in Table 3. Of the 72 negative specimens, 69 yielded negative results, and 40 out of the 42 positive samples showed positive results. The performance of “CrAg Lateral Flow Assay” was identical with that described in the kit instructions. The sensitivity and specificity of LF-RPA assay and “CrAg Lateral Flow Assay” were 95.2% (95% CI: 83.84–99.42%) vs 100% (95% CI: 91.59–100%) and 95.8% (95% CI: 88.30–99.13%) vs 100% (95% CI: 95.01–100%), respectively.

**Table 3:** Estimation of diagnostic validity of LF-RPA assay with 114 CSF specimens

		Culture/India ink staining		Performance characteristics (%)			
		Positive	Negative	Se	Sp	PPV	NPV
LF-RPA	Pos	40	3	95.2^a^	95.8^b^	93.0^c^	97.2^d^
	Neg	2	69				
CrAg Lateral Flow Assay	Pos	42	0	100^e^	100^f^	100	100
	Neg	0	72				

Pos: positive; Neg: negative; Se: sensitivity; Sp: specificity; PPV: positive predictive value; NPV: negative predictive value; CI: confidence interval.

^a^ 95% CI: 83.84%-99.42%; ^b^ 95% CI: 88.30%-99.13%; ^c^ 95% CI: 81.46%-97.59%; ^d^ 95% CI: 89.91%-99.26%; ^e^ 95% CI: 91.59%-100.00%; ^f^ 95% CI: 95.01%-100.00%.

### Comparison of different DNA extraction methods

**Fig. 4:**
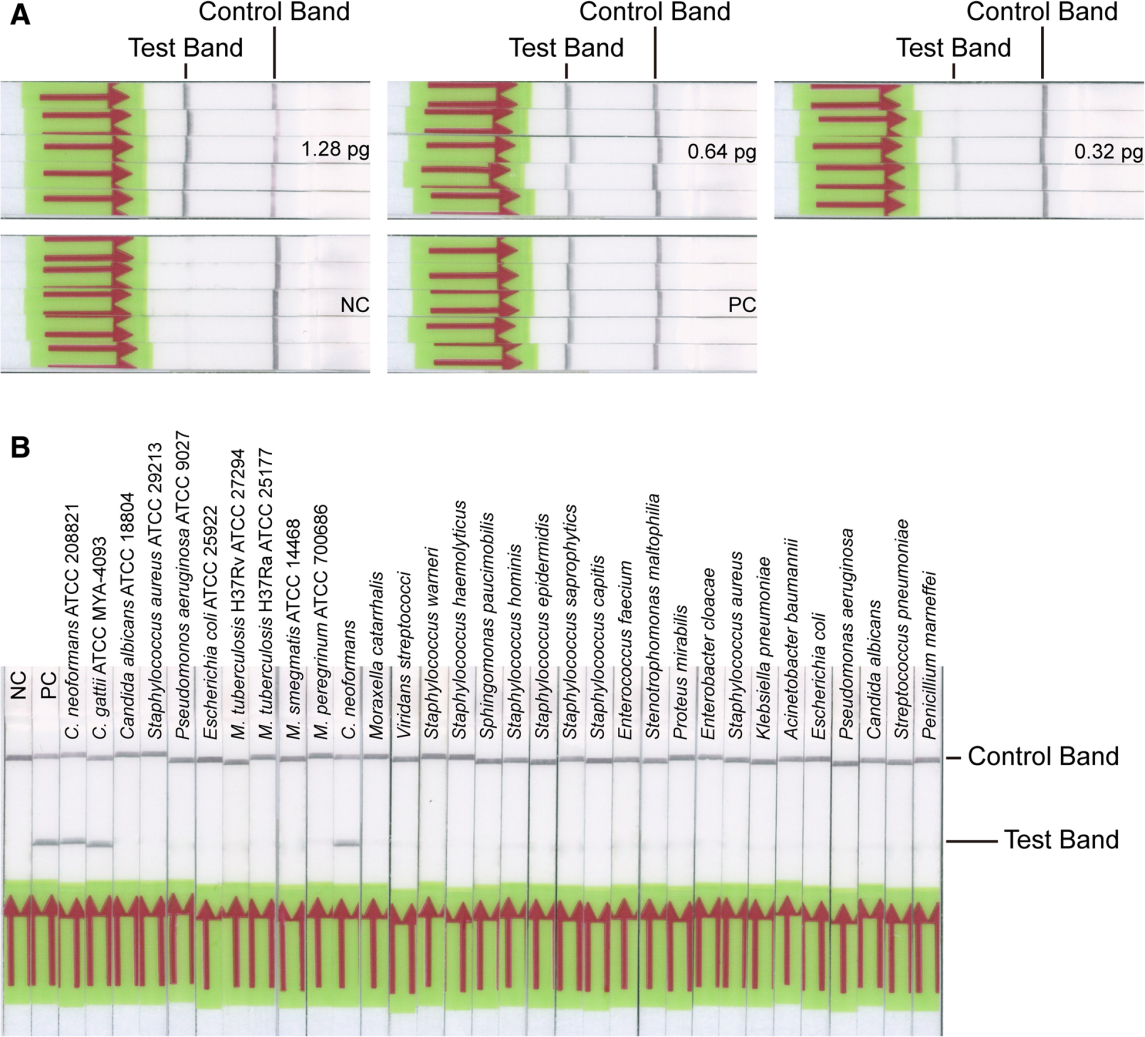
Comparison of different DNA extraction methods. Genomic DNA from “spin columns method” was used as templates to repeat the above-mentioned experiments, especially evaluation of the detection limit (**a**) and cross-reactivity analysis (**b**). The detection limit is also 0.64 pg of genomic DNA each test and no cross-reactivity was observed with any other pathogens

All the templates used in the above experiments were obtained by means of “glass beads method”. Strictly speaking, the “glass beads method” only gets cell lysates instead of the real genomic DNA. To determine whether the cell lysates affect the reaction efficiency of LF-RPA assay, we have further purified the cell lysates using “TIANamp Yeast DNA Kit”. This is “spin columns method” for genomic DNA extraction. Then, we used the DNA as templates for the similar experiments above-mentioned. As shown in Fig. 4, the detection limit of LF-RPA assay is also 0.64 pg of genomic DNA per reaction and it could only detect genomic DNA of *C. neoformans* and *C. gattii* but not other pathogens. It’s indicating that the above-mentioned different DNA extraction methods do not obviously affect the reaction efficiency of LF-RPA assay.

## Discussion

At present, there is no widely used method for detecting *Cryptococcus* spp. based on nucleic acid amplification. Now we have described a new method, lateral flow recombinase polymerase amplification assay, to detect genomic DNA of *Cryptococcus* spp. and measured its several diagnostic parameters. The detection limit of LF-RPA assay is slightly higher than that of “CrAg Lateral Flow Assay”. It is 0.64 pg of *C. neoformans* genome (approximately equal to 30 copies) per reaction and enough to detect *Cryptococcus* spp. in most natural infection (the total length of *C. neoformans* var. grubii H99 genome is about 18.9161 Mbp) (https://www.ncbi.nlm.nih.gov/genome/61?genome_assembly_id=52487). Besides, our LF-RPA method was highly specific for *Cryptococcus* spp. and had no cross-reactivity with other pathogens.

Other advantages of the system were the shorter detection time and the wider range of amplification temperature. The detection time mainly includes template preparation time, RPA amplification time, and strips incubation time. Results described here indicate that the “glass beads method”, taking about 15 min, is sufficient to meet the needs of LF-RPA assay. That is to say, the template preparation time is about 15 min. Results shown in Fig. 3a suggest that the RPA amplification time is 10 min or more. Considering the requirements of detection rapidity, sensitivity and efficiency, 15 min is really enough for target amplification [20, 25, 29]. Several earlier reports and our preliminary experiments show that 5 min is sufficient for displaying test signals [20, 25, 29–32]. In one word, the whole time of LF-RPA assay from start of template preparation to detection on strips is about 35 min. Moreover, Fig. 3b showed that the method could run well at a wide range of temperature from 25 to 45 °C, suggesting that running this system does not require additional special heating equipment [25]. Furthermore, there is no need for trained staff to interpret the results because the test results appeared as visible band can be easily read with naked eyes by untrained personnel, which saves labor costs and time [25, 29]. To summarize, the LF-RPA assay presented here is a relatively ideal method for rapid and visual detection of *Cryptococcus* spp. especially in remote regions.

Subsequently, 114 clinical specimens were used to calculate the diagnostic parameters of the LF-RPA system. Almost perfect combination of primer-probe and high amplification efficiency are the basement and guarantee of high sensitivity. Relatively higher sensitivity indicates that the LF-RPA method might be useful for clinical preliminary screening of *Cryptococcus* spp.. However, high sensitivity might also lead to higher false positive rate [33, 34]. Of course these false positive specimens might also be cross-contaminated by other positive samples or polluted by laboratory environment although there are strict antipollution measures.

At first we thought that the relatively pure template would obviously affect the detection efficiency of the system. However, this was not the case. As amplification template, both cell lysates and genomic DNA produce similar experimental results (Fig. 4). It is clearly shown that the RPA system is robust and highly tolerant [29, 30, 35].

There is still the undeniable fact that our LF-RPA system has many shortcomings. (1) The performance characteristics of our LF-RPA assay have not been established for plasma, serum, or whole blood other than cerebral spinal fluid. This will be one of our main research directions in the future. (2) Compared with other molecular detection methods, the current cost of the LF-RPA assay is relatively higher. As the yield increase, prices may decrease in the future. (3) Although the whole process of visual analysis is carried out in a dedicated class II biosafety cabinet, the possibility of cross contamination between samples is still high because of the abundant amplicons. A cross-contamination-proof cassette will be used to eliminate the need of opening the amplification tubes, thus minimizing the chance of contamination [36–38]. (4) Recently, duplex amplification detection of genomic DNA from *Francisella tularensis* and *Yersinia pestis* on only one nucleic-acid lateral flow strip has been reported [39]. It just confirms our ongoing research: duplex lateral flow assay for the simultaneous detection of *C. neoformans* and *Canidia albicans*. This is our another research direction. Although there are many methods for detecting pathogens based on RPA assays, there is still a long way before they are actually applied to clinical practice.

## Conclusions

In conclusion, the LF-RPA system described here is shown to be a sensitive, specific, and robust method for the visible, rapid, and accurate detection of *Cryptococcus* spp. in cerebral spinal fluid and might be useful for clinical preliminary screening of cryptococcal meningitis.

## Abbreviations

ATCC: American type culture collection
CM: Cryptococcal meningitis
CrAg: Cryptococcal antigen polysaccharide
CSF: Cerebral spinal fluid
ITS: Internal transcribed spacer
LF: Lateral flow
RPA: Recombinase polymerase amplification
SDA: Sabouraud dextrose agar
